# Genetic variability of *Leishmania (Leishmania) infantum* causing human visceral leishmaniasis in the Southeastern Brazil

**DOI:** 10.1590/S1678-9946202365055

**Published:** 2023-10-20

**Authors:** Vinicius Alves Lima, Renata Elen Costa Silva, Luiz Henrique Moraes Caetano Camargo, Roberto Mitsuyoshi Hiramoto, Elcio de Souza Leal, Lucia Maria Almeida Braz, José Angelo Lauletta Lindoso

**Affiliations:** 1 Universidade de São Paulo, Faculdade de Medicina, Departamento de Moléstias Infecciosas e Parasitárias, São Paulo, São Paulo, Brazil; 2 Universidade de São Paulo, Faculdade de Medicina, Instituto de Medicina Tropical de São Paulo, Laboratório de Protozoologia (LIM-49), São Paulo, São Paulo, Brazil; 3 Universidade Federal de São Paulo, Laboratório de Endocrinologia Molecular e Translacional, São Paulo, São Paulo, Brazil; 4 Instituto Adolfo Lutz, São Paulo, São Paulo, Brazil; 5 Universidade Federal do Pará, Instituto de Ciências Biológicas, Belém, Pará, Brazil; 6 Universidade de São Paulo, Faculdade de Medicina, Departamento de Medicina Preventiva, São Paulo, São Paulo, Brazil; 7 Instituto de Infectologia Emilio Ribas, São Paulo, São Paulo, Brazil

**Keywords:** Leishmania infantum, Human visceral leishmaniasis, kDNA minicircle, Sequencing, Polymorphism

## Abstract

*Leishmania infantum* is a protozoan that causes visceral leishmaniasis (VL) in the Americas and some regions of Europe. The disease is mainly characterized by hepatosplenomegaly and fever, and can be fatal. Factors related to the host and parasite can contribute to the transmission of *Leishmania* and the clinical outcome. The intraspecific genetic variability of *L. infantum* strains may be one of these factors. In this study, we evaluated the genetic variability of *L. infantum* obtained from bone marrow smear slides from patients in the Sao Paulo State, Brazil. For this, the minicircle of the kDNA hypervariable region was used as target by Sanger sequencing. By analyzing the similarity of the nucleotides and the maximum likelihood tree (Fasttree), we observed a high similarity (98%) among samples. Moreover, we identified four different profiles of *L. infantum*. In conclusion, *L. infantum* strains from Sao Paulo State, Brazil, showed low diversity measured by minicircle of the kDNA hypervariable region.

## INTRODUCTION

Visceral leishmaniasis (VL) is a parasitic disease caused by two species of *Leishmania*; *Leishmania (Leishmania) donovani*; and *Leishmania (L.) infantum (sin Leishmania (L.) chagasi)*, according to geographic region^1^, occurring autochthonously in developed and underdeveloped countries. In Brazil, VL is a zoonosis caused by *L. infantum*, transmitted mainly by *Lutzomyia longalpis* ^1^. The human disease, still endemic in the country, quickly spread to other regions and urban centers in the country, with a series of cases being observed since 1999 in the Sao Paulo State, Brazil. Currently, it is known that its expansion through the Sao Paulo State follows the route of the Marechal Rondon highway, in the west-east direction^2,3^. A spread of human visceral leishmaniasis (HVL) was noted due to the large flow of people in these highways, with the first case reported in the Northwest region of Sao Paulo State. Some factors may be related to the spread of the disease, such as deforestation, construction of new roads, and immigration^1,2^. One of the factors may be related to the parasite since different strains of *L. infantum* are observed circulating across different geographic regions^4^. Genomic plasticity can be observed in *Leishmania*, which allows the parasite to adapt to different environments, including different vertebrate hosts^5-7^. Its high genetic plasticity generates a high rate of genetic mutations, which can generate heterogeneity in *Leishmania* populations^3,8-10^. Some studies have used different targets and methodologies to measure polymorphisms in *Leishmania*. In this context, various regions and genes, such as maxicircles, minicircles, hsp 70, ssrDNA, and microsatellites, are used to detect genetic polymorphisms of *Leishmania* ^3,4,7,8,10,11^. In this study, we used the hypervariable region of the kDNA minicircle to evaluate the presence of polymorphisms of *L. infantum* in samples from the Sao Paulo State, Brazil. This approach is due to the high discriminatory capacity of the kDNA minicircle in the sense of geographical regionalization of variant genotypes, previously described in the literature^6,11,12^.

## MATERIALS AND METHODS

### Ethics statement

This study was approved by the IMT-USP Research Ethics Committee, CAEE protocol Nº: 45561115.0.0000.0065.

### Samples

Samples prepared in Giemsa-stained smears on microscope slides were obtained from 66 bone marrow aspirates from patients with visceral leishmaniasis, from January 2007 to June 2016, provided by Instituto Adolfo Lutz. The samples were from different regions from Sao Paulo State (23° 32’ 51” S 46° 38’ 19” W), located in Southeastern Brazil.

### Molecular procedures

#### DNA extraction

The smears stained with Giemsa on microscope slides were subjected to a previous hydration step to eliminate excess dyes and facilitate scraping of the fixed material, allowing greater recovery of the genetic material. They were immersed in alcohols, for 5 min, in decreasing concentrations of ethanol: 100%, 90%, 80%, 70%, ending in distilled water. After hydration, the samples were subjected to DNA extraction following the Qiagen manufacturer’s instructions and the DNA was quantified by NanoDrop 1000 (Thermo Scientific TM, USA).

#### Polymerase chain reaction

Positive samples were subjected to the MC1 and MC2 primers (5’ GTTAGCCGATGGTGGTCTTG 3’ CACCCATTTTTCCGATTTTG 3’), specific for the *Leishmania (Leishmania)* subgenus of the *donovani* complex. PCR (MC1/MC2) amplified 447bp of the partial sequence of the minicircles, following the PCR-kDNA protocol^12^. The amplified products were purified following the manufacturer recommendations of QIAquick PCR purification kit (Qiagen, Switzerland).

#### Sequencing and genetic analysis

For the sequencing reaction, the BigDye Terminator v3.1 Cycle sequencing kit (Applied Biosystems, USA) was used. Sequencing reading was performed on the ABI PRISM 3100 Genetic Analyzer (Applied Biosystems, USA). For genetic identity analysis, the results were presented in a frequency distribution of identity pairs in a graphical interface.

The similarity between Brazilian sequences and some strains of *Leishmania* spp. is indicated by color percentages, according to the scale pattern indicated in each diagram. For the phylogenetic analysis, the best model was selected according to the likelihood ratio test (LRT) implemented in the jModeltest tool. The maximum likelihood phylogenetic tree was inferred using the GTR+ gamma correction and the model proportion of invariant locations. Branch support was obtained by the approximate likelihood ratio test (aLRT) and is shown in a color scale.

## RESULTS

**Figure 1:**
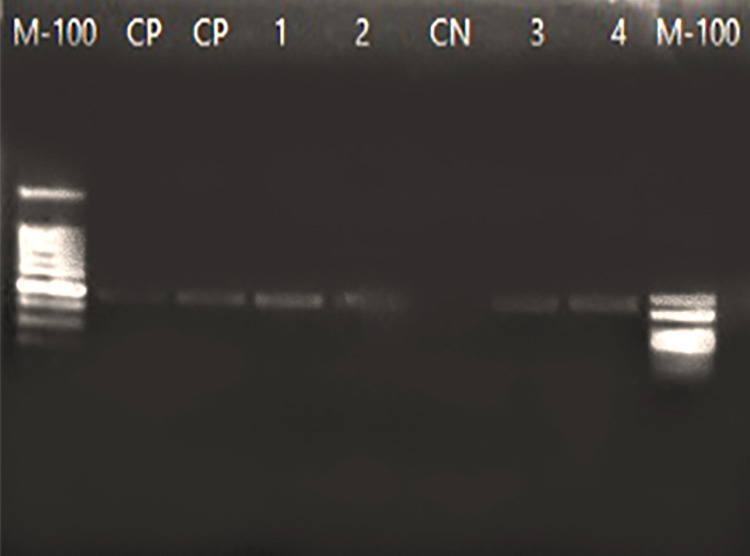
Agarose gel electrophoresis (1.8%) of amplified DNA from bone marrow smear of patients with visceral leishmaniasis after PCR-kDNA (MC1/MC2 primers). M-100 (ladder of 100bp), PC (Positive Control), 1 and 2 (promastigote DNA added to healthy individual DNA), CN (negative control), 3 and 4 (patient samples); M-100 (ladder of 100 bp).

DNA extraction from fixed and stained smears on microscope slides using the modified QIAamp Mini Kit (Qiagen, Switzerland) yielded an average of 20 ng/µL and 1.8 (260/280) as a quality reference standard. Of the 66 screened samples, 22 were amplified and were positive for *L. infantum* DNA by PCR-kDNA (MC1/MC2), which corresponds to a fragment of 447 bp (Figure 1).

**Figure 2:**
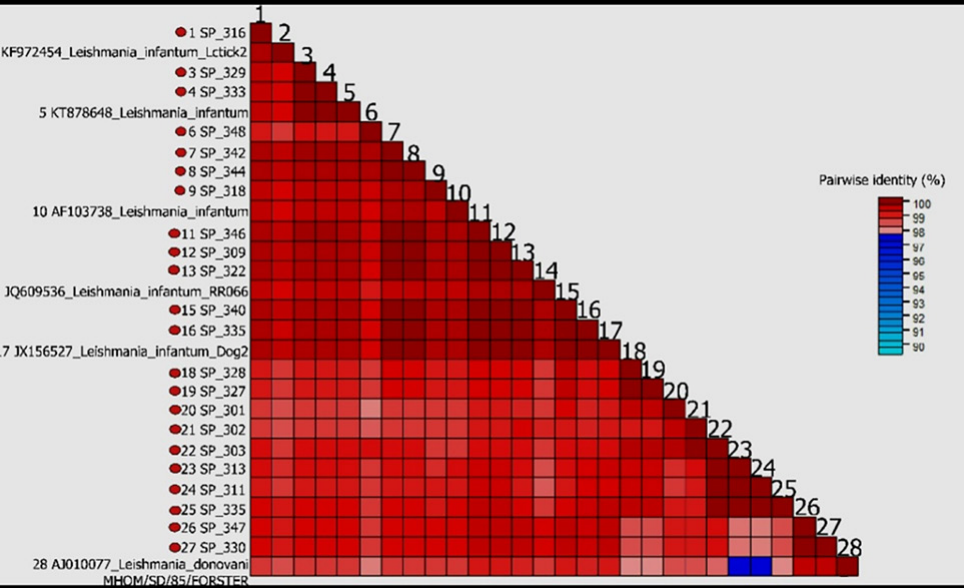
Nucleotide similarity of the hypervariable region of the *Leishmania* kinetoplast. Diagrams indicate similarity between the Brazilian sequences and some strains of *Leishmania* spp. The similarity percentages are indicated by colors according to the scale pattern indicated in each diagram.

When analyzing the nucleotide similarity of the kDNA minicircle, the Sao Paulo State samples showed homogeneity, with 98% of similarity among them when compared with isolates available on GenBank (Figure 2).

**Figure 3:**
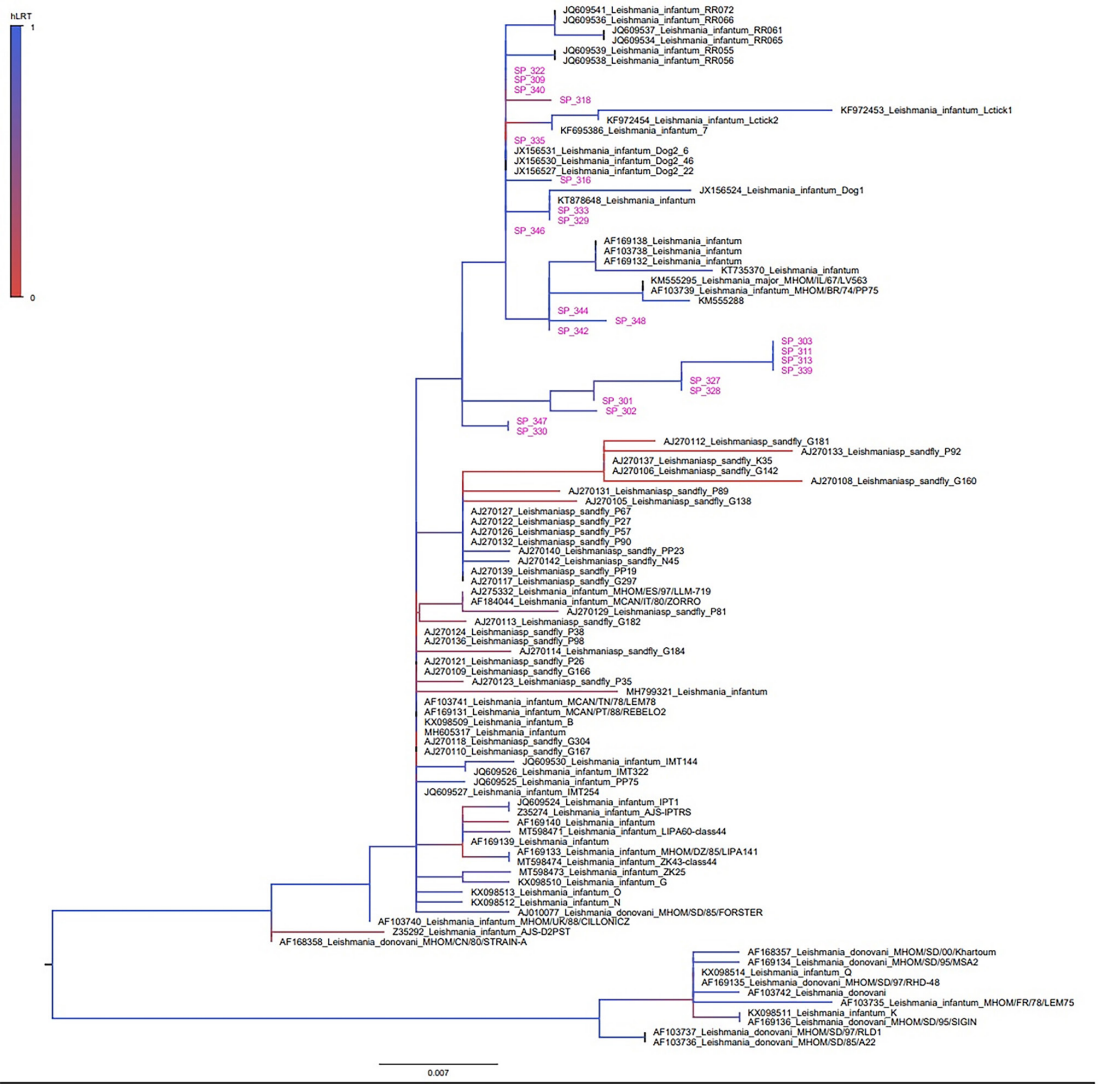
Maximum likelihood tree of the hypervariable region (447pb) of the *Leishmania* kinetoplast. The tree was inferred in Fasttree software program using GTR+ gamma correction and the model proportion of invariant locations selected by the jModeltest tool. Branch support was obtained by the approximate likelihood ratio test (aLRT) and is shown in a color scale. The sequences generated in this study are highlighted in gray.

According to the maximum likelihood phylogenetic tree of the kDNA minicircle, we observed that the samples from Sao Paulo presented a distribution in a smaller branch, divided into four slightly different profiles. They are more distant from other GenBank isolates from sandflies and Old-World dogs (Figure 3).

## DISCUSSION

Polymorphisms in trypanosomatids, more specifically in the genus *Leishmania* spp., have been reported in the Americas, mainly in Brazil. Some of the studies detected the presence of polymorphisms and genotypes that cause tegumentary leishmaniasis^13^. However, there are few works of genotyping and identification of polymorphisms of *Leishmania* species that cause visceral leishmaniasis. Most of these studies used culture isolates and, therefore, *Leishmania* in its promastigote morphology. Few studies used amastigotes obtained directly from the bone marrow, as was one of the focuses of our work^4,8,11^. Recent studies that have investigated polymorphisms showed a considerable degree of homogeneity between populations of *L. infantum* in humans, dogs, and sandflies, circulating both in the Old and New World^5,14-17^. These findings also contrast with different screening targets, such as *hsp*70, maxicircle, and microsatellite panel that distinguished distinct profiles and dispersal routes. However, they showed aneuploidy and low variability among isolates^7,9,18,19^. Few studies presented some distinction and characterization of these populations present in *L. infantum*; however, they were based on culture isolates^5,7,10,12^. Isolates obtained from culture of *Leishmania* are considered an inconvenient bias for the interpretation of results, according to Tomás-Pérez *et al*.^20^. On the other hand, Cupollilo *et al.* ^7^ showed similarity in paired samples from tissue and culture by using MLMT by microsatellites. Our study detected homogeneity among the strains of *L. infantum* obtained from the bone marrow. When our sequences were grouped and compared with different strains from the GenBank, a uniform distribution was observed and most of them presented a very high level of similarity between them (Figure 2 and 3). Therefore, it was not possible to consider a great genetic population diversity of *L. infantum* in Sao Paulo State using our methodology. To investigate the presence of polymorphisms in *L. infantum,* we investigated some studies that chose kDNA as a target by PCR, i.e., the *Leishmania* DNA sequence that appears in the greatest number of times and repeatedly^8,11,12,16^.

However, differently from other authors^8,11,12^, we evaluated the genetic variability directly from the parasite DNA in the smears, rather than from culture isolates. The cited authors used PCR-RFLP to target the kDNA region and identify the predominant polymorphisms and genetic profiles of *Leishmania* spp. by performing a previous culture and concentration of parasites in the promastigote form. Our studies demonstrated genetic variability among samples, however, they did not confirm the presence of polymorphisms in the same kDNA target region. Moreover, other recent studies^4,15,16^ found a high degree of homogeneity when compared to other molecular techniques and geographic region. Furthermore, the profiles obtained were grouped according to their geographic region^4,12^. Using Sanger sequencing (kDNA region), four possible different clades were found with the samples distributed randomly. Nunes *et al*.^17^ also observed homogeneity analyzing 29 isolates of *L. infantum* from the bone marrow of naturally infected dogs by molecular characterization using *hsp*70, mpi, and ITS1. Solana *et al*.^18^ showed that the maxicircle sequence can be used as a robust molecular marker for phylogenetic analysis and species typing within kinetoplastids, which also shows potential to discriminate intraspecific variability. Moreover, it is important to emphasize the difference between *L. infantum* strain from the New and Old World since the latter shows a variety of *L. infantum* zymodemas^11,12^. More modern techniques, such as NGS-based are necessary to better understand the genetic structure of *L. infantum* from the New World, as well as phenotypic assays to understand how this diversity can influence the disease outcome. However, it is noteworthy that our hypervariable sequence of minicircles methodology (MC1/MC2) was low cost, effective, practical, and quick in elucidating the results.

## CONCLUSION

In conclusion, we highlight that our samples presented low diversity when the markers we selected were used in direct samples (amastigotes) of bone marrow. However, our data are limited by the sample size, and heterogeneity of *L. infantum* in Sao Paulo State, Brazil, may exist. A more detailed analysis should be performed using more than one molecular target, a consistent number of viable biological samples, and multiple *L. infantum* isolates from different regions.
